# A phase II trial of small-dose bortezomib, lenalidomide and dexamethasone (sVRD) as consolidation/maintenance therapy in patients with multiple myeloma

**DOI:** 10.1007/s00280-016-3163-y

**Published:** 2016-10-13

**Authors:** Soushi Ibata, Tsutomu Sato, Hiroyuki Kuroda, Yasuhiro Nagamachi, Satoshi Iyama, Akihito Fujimi, Yusuke Kamihara, Yuichi Konuma, Masahiro Yoshida, Ayumi Tatekoshi, Akari Hashimoto, Hiroto Horiguchi, Kaoru Ono, Kazuyuki Murase, Kohichi Takada, Koji Miyanishi, Masayoshi Kobune, Yasuo Hirayama, Junji Kato

**Affiliations:** 1 Department of Medical Oncology and Hematology, Sapporo Medical University School of Medicine, South-1 West-16, Chuo-ku, Sapporo, Japan; 2 Gastroenterology and Hematology/Clinical Oncology, Internal Medicine, Steel Memorial Muroran Hospital, Muroran, Japan; 3 Department of Hematology, Kiyota Hospital, Sapporo, Japan; 4 Department of Hematology and Oncology, Oji General Hospital, Tomakomai, Japan; 5 Department of Hematology and Oncology, Asahikawa Red Cross Hospital, Asahikawa, Japan; 6 Division of Internal Medicine, Higashi Sapporo Hospital, Sapporo, Japan

**Keywords:** Multiple myeloma, Consolidation/maintenance, Bortezomib, Lenalidomide, Dexamethasone, VRD

## Abstract

**Purpose:**

Consolidation/maintenance therapy induces deep remission in patients with multiple myeloma (MM); however, the most suitable regimen has been under investigation. The combination therapy with bortezomib, lenalidomide and dexamethasone (VRD) is a powerful regimen for relapsed/refractory as well as newly diagnosed MM as an induction therapy. However, severe adverse events (AEs) may become a problem when VRD is introduced without dose reduction as a consolidation/maintenance therapy.

**Methods:**

In this single-arm phase II study, we evaluated the efficacy of small-dose VRD regimen (sVRD) in the consolidation/maintenance setting. Sixteen patients who had partial response (PR) or better after any induction therapy were enrolled. Patients received at least six 28-day cycles of subcutaneous bortezomib (1.3 mg/m^2^ on days 1 and 15), lenalidomide (10 mg on days 1–21) and dexamethasone (40 mg on days 1, 8, 15 and 22).

**Results:**

The overall response rate and the complete response (CR) rate were 100 and 43.8 %, respectively. In particular, one patient with CR and two patients with very good PR at enrollment achieved stringent CR during 6 courses of sVRD. With a median follow-up time of 29.4 months, the median progression-free survival (PFS) and overall survival (OS) were not reached, while the PFS and OS rates at 2.5 years were 66.6 and 77.3 %, respectively. Univariate analysis demonstrated that disease progression as a reason for discontinuation of sVRD had a negative impact on OS. There were no grade 3 or 4 hematologic or nonhematologic AEs.

**Conclusion:**

Our sVRD regimen as a consolidation/maintenance therapy was highly effective and well tolerable.

## Introduction

Over the past 10 years, the median overall survival (OS) of patients with multiple myeloma (MM) has considerably increased due to the use of autologous hematopoietic stem cell transplantation (HSCT) and the introduction of the immunomodulatory drugs, thalidomide and lenalidomide, and the proteasome inhibitor, bortezomib, in transplant-eligible and transplant-ineligible patients [1].

In order to consolidate and maintain the outcome after induction therapy with these novel agents, consolidation/maintenance therapy has been an attractive choice. Consolidation (two to four cycles of combination therapies) and maintenance (continuous therapy, usually with single agents, until the time of disease progression) are commonly used in clinical practice after induction therapy, although no specific guidelines are available [2].

There have been many trials to support the use of consolidation/maintenance to maintain the response achieved after autologous HSCT or conventional treatments and to improve patient survival with single agent or combination therapy: thalidomide [3–8], lenalidomide [9–12], bortezomib [13], bortezomib plus thalidomide [14, 15] and bortezomib, thalidomide plus dexamethasone [16, 17]. However, no definitive information is available regarding which drug or which combination of drugs is the most favorable for consolidation/maintenance.

Concerning this matter, Kikuchi et al. [18] published an informative study using in vitro isobologram analysis. They demonstrated that lenalidomide has strong combined effects with bortezomib on myeloma cells in the presence of stromal cells. The bortezomib-induced up-regulation of CCAAT/enhancer-binding protein homologous protein (CHOP), a pro-apoptotic transcription factor, was readily enhanced by lenalidomide in contact with stromal cells. Their findings are compatible with the report that the overall response rate (ORR) (i.e., very good partial response or better) of the combination of bortezomib, lenalidomide and dexamethasone (VRD) was higher than those of bortezomib, doxorubicin and dexamethasone (PAD), bortezomib, thalidomide and dexamethasone (VTD), or cyclophosphamide, bortezomib and dexamethasone (CVD) in newly diagnosed myeloma patients [19].

The combination regimen of VRD was first evaluated in patients with relapsed or relapsed/refractory MM in a phase I, dose-escalation study by Richardson et al. [20]. Then, they reported a phase II study to evaluate the efficacy and safety of VRD in the same relapsed or relapsed/refractory setting [21]. Also in a frontline setting, they reported favorable toxicity and promising response and survival of patients treated with the VRD regimen in a phase I/II study [22]. Some other reports confirmed the efficacy of the VRD regimen as a frontline [23] or second-line treatment [24, 25].

Especially, Roussel et al. [23] evaluated the efficacy of three courses of the VRD regimen as an induction treatment for previously untreated patients; their VRD regimen consisted of 3-week cycles of intravenous bortezomib 1.3 mg/m^2^ on days 1, 4, 8 and 11; oral lenalidomide 25 mg on days 1 to 14; and oral dexamethasone 40 mg on days 1, 8 and 15. They reported that the ORR at the completion of induction therapy was 58 %.

In consideration of this high efficacy, the combination of bortezomib, lenalidomide and dexamethasone is attractive for consolidation/maintenance treatment; however, adverse events (AEs) were not negligible with the full dosage of their VRD regimen. The most common toxicities with the VRD regimen were neurologic and hematologic, including grade 1–2 sensory neuropathy (55 %), grade 3–4 neutropenia (35 %) and thrombocytopenia (13 %) [23].

Therefore, we conducted a phase II study reported herein evaluating the efficacy and safety of small-dose VRD (sVRD) in the consolidation/maintenance setting.

## Methods

### Study design and objective

The aim of this multicenter, open-label, single-arm, phase II study was to determine the efficacy and safety of sVRD in Japanese patients with MM in the consolidation/maintenance setting. The primary end point of this study was the best ORR during 6 courses of sVRD. Secondary end points included progression-free survival (PFS), OS and safety. This study was conducted according to the Declaration of Helsinki and was approved by the institutional review board of each participating center. The institutional review board-approved consent form was signed by all patients before participating in this study. This trial is registered at www.umin.ac.jp (#UMIN8236).

### Patients

Eligible patients were age ≥20 and ≤80 years, with measurable symptomatic MM. Patients must have received at least 1 prior regimen and achieved at least a partial response (PR) by the International Myeloma Working Group (IMWG) Uniform Response Criteria. Other eligibility criteria included Eastern Cooperative Oncology Group (ECOG) performance status (PS) of 0–2, and an expected survival of more than 3 months. Adequate pulmonary, cardiac, renal and hepatic functions were required.

### Treatment

Patients received subcutaneous bortezomib (1.3 mg/m^2^ on days 1 and 15), oral lenalidomide (10 mg on days 1–21) and oral dexamethasone (40 mg on days 1, 8, 15 and 22). The course was repeated every 4 weeks for 6 cycles. Patients with at least a PR at the end of cycle 6 could continue sVRD treatment. Patients discontinued therapy if they experienced progressive disease (PD) or unacceptable toxicity, if no more additional benefits could be expected or if the patient/investigator decided to discontinue therapy for any reason. Dose adjustments were permitted based on grade 3 or 4 AEs or based on an investigator’s decision; bortezomib could be reduced from 1.3 to 1.0 mg/m^2^, lenalidomide from 10 to 5 mg/day and dexamethasone from 40 to 20 mg/day. If a similar severity of toxicity occurred at the reduced dose, study treatment was discontinued. Antiviral prophylaxis, bisphosphonates, aspirin thromboprophylaxis and erythropoietic agents were permitted during the study. Granulocyte colony-stimulating factor was also allowed.

### Assessment of efficacy

Response assessments were conducted before enrollment and after each course of sVRD treatment. The ORR was defined as the proportion of patients whose best overall response was either a stringent complete response (sCR), complete response (CR), very good PR (VGPR) or PR based on responses as assessed using IMWG Uniform Response Criteria. sCR, CR, VGPR and PR required two consecutive assessments made at any time before progression or initiation of any new therapy. Patients were followed for disease progression and OS for up to 3 years after discontinuation or completion of therapy.

### Assessment of safety

AEs were assessed at each visit and were graded according to National Cancer Institute Common Terminology Criteria (NCI-CTC) for AEs (Version 4.0). Data were collected until 30 days after the last dose of study drug, except for secondary primary malignancies (SPM) (which were assessed all along during follow-up). SPM was defined as any malignancy observed after introduction of sVRD treatment.

### Statistical methods

The median follow-up time was estimated using the reverse Kaplan–Meier method. PFS was calculated as the time from the start of treatment to the first documentation of PD or death if the patient died as a result of any cause before progression. OS was calculated as the time from the start of treatment to death. The Kaplan–Meier method was used to estimate the survival distribution. Univariate survival analysis was performed using the Kaplan–Meier method. The significance of differences in survival curves was assessed with the log-rank test. Multivariable analysis (Cox proportional hazards regression model) of OS was carried out on all covariates that showed a significant association with OS in univariate analysis. All analyses were conducted using GraphPad Prism version 5.0 (GraphPad Software, La Jolla, CA) and EZE (Saitama Medical Center, Jichi Medical University; http://www.jichi.ac.jp/saitama-sct/SaitamaHP.files/statmedEN.html) [26].

## Results

### Patients and treatments

From June 2012 until November 2013, 16 MM patients were enrolled at 4 sites in Japan. The data cutoff date for this analysis was March 10, 2015. Patient demographics and baseline characteristics are summarized in Table 1. The median age was 67 years (range 53–78 years); 12 patients (75.0 %) were >65 years. Eleven patients (68.8 %) were male. The median time from diagnosis to enrollment was 17 months (range 4–95 months). ECOG PS was 0 in 50.0 %, 1 in 37.5 % and 2 in 12.5 % of the patients. They had either IgG (62.5 %) or IgA (37.5 %) myeloma and had either Kappa (81.3 %) or Lambda (18.7 %) light chain. After restaging at the time of enrollment, 81.3 % of patients had stage I and 18.7 % stage II according to the classification system of the International Staging System (ISS), and 87.5 % had stage I and 12.5 % stage III according to the system of Durie and Salmon (D-S). Fluorescence in situ hybridization (FISH) performed at the time of diagnosis showed that 1 (9.1 %) out of 11 patients had del 17, 4 (44.4 %) out of 9 del 13 and 3 (42.9 %) out of 7 *t*(4;14). The induction therapies before enrollment in this study were as follows: 87.5 % of patients were treated with dexamethasone, 81.3 % bortezomib, 43.8 % lenalidomide, 25.0 % doxorubicin, 18.8 % melphalan and 12.5 % cyclophosphamide. 43.8 % of patients had undergone radiation therapy. 25.0 % of patients had undergone at least one HSCT.

**Table 1:** Characteristics of the patients with MM who received the sVRD regimen

Characteristic	All patients (*n* = 16)
Age [mean (range)]	67 (53–78)
Male sex [*n* (%)]	11 (68.8)
Mean time from diagnosis to enrollment [months (range)]	17 (4–95)
PS [*n* (%)]	
0	8 (50.0)
1	6 (37.5)
2	2 (12.5)
Type of myeloma [*n* (%)]	
IgG	10 (62.5)
IgA	6 (37.5)
Kappa	13 (81.3)
Lambda	3 (18.7)
ISS stage at enrollment [*n* (%)]	
I	13 (81.3)
II	3 (18.7)
III	0 (0)
D–S stage at enrollment [*n* (%)]	
I	14 (87.5)
II	0 (0)
III	2 (12.5)
Cytogenetic abnormalities^a^ [*n* (%)]	
del 17	1/11 (9.1)
*t*(14;16)	0/7 (0)
del 13	4/9 (44.4)
*t*(4;14)	3/7 (42.9)
Induction regimen [*n* (%)]	
Any use of dexamethasone	14 (87.5)
Any use of bortezomib	13 (81.3)
Any use of lenalidomide	7 (43.8)
Any use of doxorubicin	4 (25.0)
Any use of melphalan	3 (18.8)
Any use of cyclophosphamide	2 (12.5)
Radiation	7 (43.8)
HSCT	4 (25.0)

*ISS* International Staging System, *D–S* Durie and Salmon, *HSCT* hematopoietic stem cell transplantation

^a^Data were obtained by fluorescence in situ hybridization (FISH)

### Duration of treatment

All patients could complete 6 courses of sVRD treatment (Table 2). The median duration of sVRD treatment was 8.0 courses (range 6–28 courses), with 56.3 % (*n* = 9) and 25.0 % (*n* = 4) undergoing >6 and >12 cycles, respectively. At the data cutoff date, all 16 patients had discontinued treatment. The reasons for treatment discontinuation were completion of 6 courses (*n* = 7, 43.8 %), disease progression (*n* = 3, 18.8 %), SPM of acute lymphoblastic leukemia (ALL) (*n* = 1, 6.3 %), AE of grade 2 pneumonia (*n* = 1, 6.3 %) or other (patient refusal or physician preference) (*n* = 4, 25.0 %).

**Table 2:** Treatment duration of the sVRD regimen

	All patients (*n* = 16)
Completion of 6 courses [*n* (%)]	16 (100)
Mean treatment duration [courses (range)]	8.0 (6–28)
Reason for discontinuation [*n* (%)]	
Completion of 6 courses	7 (43.8)
Disease progression	3 (18.8)
Second primary malignancy (acute lymphoblastic leukemia)	1 (6.3)
Adverse events (pneumonia)	1 (6.3)
Others	4 (25.0)

### Response

All 16 patients were response-evaluable. The ORR during 6 courses of sVRD treatment is shown in Table 3. 43.8 % had an sCR, 0 % CR, 6.3 % VGPR, 50.0 % PR and 0 % PD. The ORR and CR rate (i.e., at least CR) were 100 and 43.8 %, respectively. In detail, at enrollment, 4 patients were determined to be in sCR, 1 in CR, 2 in VGPR and 9 in PR. During 6 courses of sVRD, 4 patients with sCR at enrollment remained in sCR. One patient with CR and 2 with VGPR achieved sCR. In 9 patients with PR, 1 achieved VGPR, but 8 remained in PR. Nevertheless, 2 out of 8 patients with PR after 6 courses of sVRD finally achieved VGPR or sCR after a total of 18 or 24 courses of sVRD, respectively. Status at enrollment of a patient with del(17p) was PR, and his best overall response was the same PR. Table 4 demonstrates that all four patients (No. 5–8) who obtained deeper response during 6 courses of sVRD were treated without lenalidomide in induction therapies.

**Table 3:** Best overall response during 6 courses of sVRD

Status at enrollment	Best overall response				
	sCR	CR	VGPR	PR	PD
sCR (*n* = 4)	4	0	0	0	0
CR (*n* = 1)	1	0	0	0	0
VGPR (*n* = 2)	2	0	0	0	0
PR (*n* = 9)	0	0	1	8	0
Total (*n* = 16) [*n* (%)]	7 (43.8)	0 (0)	1 (6.3)	8 (50.0)	0 (0)

*sCR* stringent complete response, *CR* complete response, *VGPR* very good partial response, *PR* partial response, *PD* progressive disease

**Table 4:** Best overall response and induction regime

Patient no.	Status at enrollment	Best overall response	Induction regimen							
			Bor	Len	Dex	Dox	Mel	CY	RT	HSCT
1	sCR	sCR		✓	✓				✓	✓
2	sCR	sCR	✓	✓	✓					✓
3	sCR	sCR	✓		✓					
4	sCR	sCR	✓	✓	✓				✓	
5	CR	sCR	✓		✓					
6	VGPR	sCR	✓	○	✓			✓		
7	VGPR	sCR	✓		✓				✓	
8	PR	VGPR	✓		✓		✓			
9	PR	PR		✓	✓	✓				✓
10	PR	PR	✓				✓			
11	PR	PR	✓	✓	✓	✓			✓	
12	PR	PR	✓				✓			
13	PR	PR	✓	✓	✓	✓			✓	
14	PR	PR		✓	✓	✓				✓
15	PR	PR	✓		✓				✓	
16	PR	PR	✓		✓			✓	✓	
Total (*n* = 16)	16	16	13	7	14	4	3	2	7	4

*Bor* bortezomib, *Len* lenalidomide, *Dex* dexamethasone, *Dox* doxorubicin, *Mel* melphalan, *CY* cyclophosphamide, *RT* radiation, *HSCT* hematopoietic stem cell transplantation

### PFS and OS

**Fig. 1:**
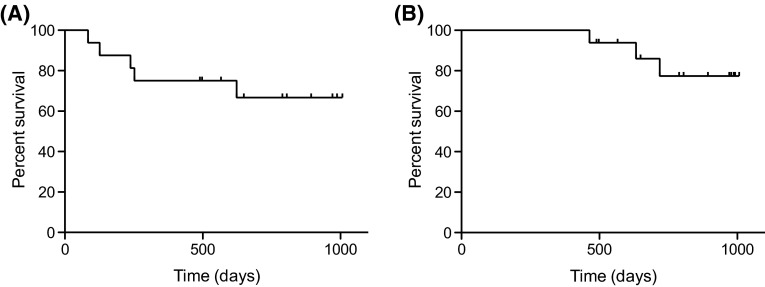
Kaplan–Meier curves of PFS and OS of MM patients who received the sVRD regimen. **a** PFS (the median PFS was not reached). **b** OS (the median OS was not reached)

With a median follow-up time of 29.4 months (range 16.1–33.1 months), 13 out of 16 patients are still alive, 11 of whom are progression-free for a maximum of 33.1 months. Three patients died and their cause of death was disease progression in all cases. One of these three patients who died had SPM of myelodysplastic syndrome (MDS). One had chromosomal abnormality of del(17p). It is noteworthy that the three patients who discontinued sVRD treatment due to PD were the same three patients who died in spite of various post-study therapies. Figure 1 shows the Kaplan–Meier estimates of PFS and OS. The median PFS was not reached, and the 2.5-year PFS was 66.6 % (95 % confidence interval [CI] 36.9–84.8 %). The median survival time (MST) was also not reached, and the 2.5-year survival rate was 77.3 % (95 % CI 44.3–92.2 %).

### Univariate analysis

When analyzing prognostic factors for OS, univariate analysis using the log-rank test confirmed well-known prognostic parameters such as PS (*P* = 0.00569), albumin (*P* = 0.0000323), ISS (*P* = 0.0123) and del 17 (*P* = 0.00157) to be of prognostic relevance in our patient cohort (Table 5). Regarding best overall response during 6 courses of sVRD, OS was negatively influenced by PR compared with sCR, CR or VGPR (*P* = 0.0334). Furthermore, disease progression as a reason for discontinuation of sVRD had a negative impact on OS in comparison with other reasons (*P* = 0.0000374). These results indicated that refractoriness to the sVRD regimen could not be easily rescued by any post-study therapies. Nevertheless, none of the variables selected on univariate analysis was an independent prognostic marker for OS in the Cox proportional hazards regression model since the number of patients for multivariate analysis was small.

**Table 5:** Univariate analysis (log-rank test) of prognostic factors for overall survival

Characteristic	Number of cases	*P* value
Sex		
Male	11	0.27
Female	5	
Age (year)		
>67	7	0.766
≤67	9	
PS		
≥2	2	0.00569
<2	14	
Beta-2 microglobulin (mg/L)		
≥2.5	3	0.507
<2.5	13	
Albumin (g/dL)		
<3.5	2	0.0000323
≥3.5	14	
ISS stage at enrollment		
II, III	3	0.0123
I	13	
del 13		
Yes	4	0.371
No	5	
*4(4;14)*		
Yes	3	0.386
No	4	
del 17		
Yes	1	0.00157
No	10	
Status at enrollment		
PR	9	0.0731
sCR, CR, VGPR	7	
Best overall response during 6 courses		
PR	8	0.0334
sCR, CR, VGPR	8	
Reason for discontinuation		
Disease progression	3	0.0000374
Others	13	
Second primary malignancy		
Yes	2	0.296
No	14	

### Safety

One dose modification of dexamethasone from 40 to 20 mg/day was required because of grade 2 hypertension after the 3rd course of sVRD. One patient had discontinued all study drugs because of grade 2 pneumonia after 6 courses of sVRD. AEs are summarized in Table 6. Grade 2 sensory neuropathy was reported in two patients; however, neuropathy occurred in each patient during their induction therapies and lasted thereafter with no worsening. Grade 2 neutropenia occurred in 2 patients, pneumonia in 2, thrombocytopenia in 1, hypertension in 1, constipation in 1 and anemia in 1. There were no grade 3 or 4 hematologic or nonhematologic AEs.

**Table 6:** Adverse events

Adverse events	Grade 2 *n* (%)	Grade 3 *n* (%)	Grade 4 *n* (%)
Sensory neuropathy	2 (12.5)^a^	0 (0)	0 (0)
Neutropenia	2 (12.5)	0 (0)	0 (0)
Pneumonia	2 (12.5)	0 (0)	0 (0)
Thrombocytopenia	1 (6.3)	0 (0)	0 (0)
Hypertension	1 (6.3)	0 (0)	0 (0)
Constipation	1 (6.3)	0 (0)	0 (0)
Anemia	1 (6.3)	0 (0)	0 (0)

^a^Preexisting neuropathy with no worsening

### Secondary primary malignancies

After enrollment, 2 new hematologic malignancies, i.e., ALL in one patient and MDS in another patient, were diagnosed (Table 7). The time from diagnosis to enrollment was 38 or 60 months in the patient with ALL or MDS, respectively. The former patient was pretreated with vincristine, adriamycin and dexamethasone (VAD) and autologous HSCT (ASCT) and then underwent 18 courses of sVRD treatment. He discontinued sVRD due to the occurrence of ALL. He has been alive after the standard induction/consolidation chemotherapy for ALL and allogeneic HSCT. The latter patient was pretreated with VAD and bortezomib plus dexamethasone (Bd) and then underwent sVRD treatment. After the 7th course, the sVRD regimen was stopped due to disease progression. The combination regimen of melphalan, prednisone and lenalidomide (MPR) was introduced as a post-study therapy, and a gradual dose reduction of melphalan was needed because of progressive pancytopenia. The diagnosis of MDS was made based on the findings of bone marrow aspiration. He finally died of PD.

**Table 7:** Second primary malignancies (SPMs)

SPM	*n* (%)	Time to enrollment (months)	sVRD (courses)	Prior therapies
Acute lymphoblastic leukemia	1 (6.3)	38	18	VAD, ASCT
Myelodysplastic syndrome	1 (6.3)	60	7	VAD, Bd

*VAD* vincristine, adriamycin and dexamethasone, *ASCT* autologous hematopoietic stem cell transplantation, *Bd* bortezomib plus dexamethasone

## Discussion

Three previous reports investigated the regimen of bortezomib–lenalidomide–dexamethasone in the consolidation/maintenance setting [21, 23, 27] (Table 8). In the report by Roussel et al. [23], previously untreated patients were treated with the VRD regimen, as an induction therapy for three cycles and a consolidation therapy for two cycles after ASCT. Nooka et al. [27] treated previously untreated patients with the VRD regimen as a maintenance therapy after ASCT for three years. Richardson et al. [21] administered the VRD regimen in relapsed/refractory patients, as a re-induction therapy for eight cycles and a maintenance therapy until PD. In our trial, patients with at least PR after any induction therapy were enrolled to receive our sVRD regimen as a consolidation/maintenance therapy for at least 6 cycles.

**Table 8:** VRD regimens used for consolidation and/or maintenance

	Roussel et al. J Clin Oncol 2014 (23)	Nooka et al. Leukemia 2014 (27)	Richardson et al. Blood 2014 (21)	Our trial
Patients	Previously untreated	Previously untreated (high-risk)	Relapsed/ refractory	≥PR after any induction
Phases of treatment	Induction 3 cycles Consolidation (after ASCT) 2 cycles	Maintenance (after ASCT) 3 years	Induction 8 cycles Maintenance until PD	Consolidation/maintenance ≥6 cycles
Cycle length	21 days	28 days	21 days	28 days
Bortezomib	1.3 mg/m^2^ IV days 1, 4, 8, 11	1.3 mg/m^2^ IV or SC days 1, 8, 15, 22	1.0 mg/m^2^ IV days 1, 4, 8, 11	1.3 mg/m^2^ SC days 1, 15
<Average>	1.7 mg/m^2^/week	1.3 mg/m^2^/week	1.3 mg/m^2^/week	0.7 mg/m^2^/week
Lenalidomide	25 mg/body days 1–14	10 mg/body days 1–21	15 mg/body days 1–14	10 mg/body days 1–21
<Average>	17 mg/day	7.5 mg/day	10 mg/day	7.5 mg/day
Dexamethasone	40 mg/body days 1, 8, 15	40 mg/body days 1, 8, 15, 22	40 mg/body days 1, 2, 4, 5, 8, 9, 11, 12 (cycles 1–4)	40 mg/body days 1, 8, 15, 22
<Average>	40 mg/week	40 mg/week	107 mg/week	40 mg/week
PN (G1–2)	55 %	N.D.	53 %^a^	13 %^b^
NP (G3–4)	35 %	N.D.	30 %	0 %
TCP (G3–4)	13 %	N.D.	22 %	0 %
Dose modification	39 %	40 %	66 %	7 %
ORR	100 %	100 %	64 %	100 %
OS	100 % (3 years)	93 % (3 years)	65 % (2 years)	77 % (2.5 years)

*PD* progressive disease, *IV* intravenous, *SC* subcutaneous, *ASCT* autologous hematopoietic stem cell transplantation, *PR* partial response, *PN* peripheral neuropathy, *N.D.* not described, *NP* neutropenia, *TCP* thrombocytopenia, *ORR* overall response rate, *OS* overall survival

^a^6/36 patients had PN at baseline

^b^2/2 patients had PN at baseline

Even though the same terminology of VRD was used in the three previous reports, the dosages of bortezomib and lenalidomide were not the same. On average, Roussel et al. [23], Nooka et al. [27] and Richardson et al. [21] administered bortezomib at 1.7, 1.3 or 1.3 mg/m^2^/week and lenalidomide at 17, 7.5 or 10 mg/day, respectively. In our trial, bortezomib and lenalidomide were administered at 0.7 mg/m^2^/week and 7.5 mg/day, respectively. The dosages of bortezomib and lenalidomide used in our trial were the lowest compared with those reported by Roussel et al. [23], Nooka et al. [27] and Richardson et al. [21]. Improvement in tolerability and the preservation of efficacy compared with the three previous reports were important issues in our trial.

The most common toxicities related to the VRD regimen were neurologic and hematologic. Grade 1–2 peripheral neuropathy (PN) was reported in 55 or 53 % of patients by Roussel et al. [23] and Richardson et al. [21], respectively. In our trial, two patients (13 %) experienced grade 2 PN; however, their neuropathy was due to prior usage of bortezomib with no worsening after enrollment in our study. A lower occurrence of PN possibly reflects the lower dosage of bortezomib at 0.7 mg/m^2^/week. In addition, subcutaneous administration of bortezomib instead of intravenous injection may reduce the occurrence of PN since it is well known that PN of any grade was significantly less common with subcutaneous than with intravenous administration [28, 29]. As for hematologic toxicities, grade 3–4 neutropenia (NP) (35 or 30 %) and thrombocytopenia (TCP) (13 or 22 %) were reported by Roussel et al. [23] and Richardson et al. [21], respectively. In our trial, there were no cases of grade 3–4 NP and TCP, possibly reflecting the lower dosage of lenalidomide at 7.5 mg/day on average.

Furthermore, the tolerability of each VRD regimen could be evaluated by the necessity of dose modification. In the reports by Roussel et al. [23], Nooka et al. [27] and Richardson et al. [21], at least one dose modification among bortezomib, lenalidomide and dexamethasone was required in 39, 40 or 66 % of patients, respectively. On the other hand, dose modification was required in only one patient (7 %) in our trial: The dose of dexamethasone was reduced from 40 to 20 mg/week due to grade 2 hypertension after the 3rd course of sVRD. Especially, in the report by Roussel et al. [23], patients were previously untreated and relatively young (range 33–65 years) compared with our patients (range 53–78 years); however, almost 40 % of patients could not complete five courses with their dosage of bortezomib (1.7 mg/m^2^/week) and lenalidomide (17 mg/day). All of our patients could complete six courses of our sVRD regimen. Taking AEs and dose modification into consideration, the dosage of bortezomib (0.7 mg/m^2^/week) and lenalidomide (7.5 mg/day) in our trial might be well rationalized.

As for the efficacy of each VRD regimen, the ORR could be comparable. In the reports by Roussel et al. [23], Nooka et al. [27] and Richardson et al. [21], the ORR was 100, 100 or 64 %, respectively. Further, OS was 100 % (3 years), 93 % (3 years) or 65 % (2 years), respectively. The ORR and OS in the report by Richardson et al. [21] were lower than those in the reports by Roussel et al. [23] and Nooka et al. [27] since the patients in the study of Richardson et al. [21] were relapsed/refractory. In our trial, the ORR and OS were 100 and 77 % (2.5 years), respectively. Needless to say, it is difficult to precisely compare the ORR and OS of our trial with those of other three reports because of many biases. Nevertheless, it can be speculated that the low dosage of bortezomib and lenalidomide in our trial did not necessarily result in decreased efficacy. We conclude that the dosage of bortezomib and lenalidomide in our sVRD regimen may be able to reduce AEs and have preserved efficacy simultaneously in the consolidation/maintenance setting.

In conclusion, our sVRD regimen as a consolidation/maintenance therapy was well tolerable and highly effective in patients with MM who achieved at least PR after any induction therapy. These results seem comparable to those of the other VRD regimens previously published [21, 23, 27] and hence support the rationale for our ongoing phase II study of the sVRD regimen in previously untreated transplant-ineligible patients with MM.
